# Supplementary material for: Non-productive angiogenesis disassembles Aß plaque-associated blood vessels
Source: Nat Commun. 2021 May 25;12:3098. doi: 10.1038/s41467-021-23337-z (PMC8149638; doi:10.1038/s41467-021-23337-z)
Supplement: Supplementary file 1 — Supplementary Information [file 41467_2021_23337_MOESM1_ESM.pdf]

Non-productive angiogenesis disassembles Aß plaque-associated blood vessels

Maria I. Alvarez-Vergara, Alicia E. Rosales-Nieves, Rosana March-Diaz, Guiomar Rodriguez-Perinan, Nieves Lara-Urena, Clara Ortega-de San Luis, Manuel A. Sanchez-Garcia, Miguel Martin-Bornez, Pedro Gómez-Gálvez, Pablo Vicente-Munuera, Beatriz Fernandez-Gomez, Miguel A. Marchena, Andrea S. Bullones-Bolanos, Jose C. Davila, Rocio Gonzalez-Martinez, Jose L. Trillo-Contreras, Ana C. Sanchez-Hidalgo, Raquel del Toro, Francisco G. Scholl, Eloisa Herrera, Martin Trepel, Jakob Körbelin, Luis M. Escudero, Javier Villadiego, Miriam Echevarria, Fernando de Castro, Antonia Gutierrez, Alberto Rabano, Javier Vitorica, and Alberto Pascual

Supplementary Information

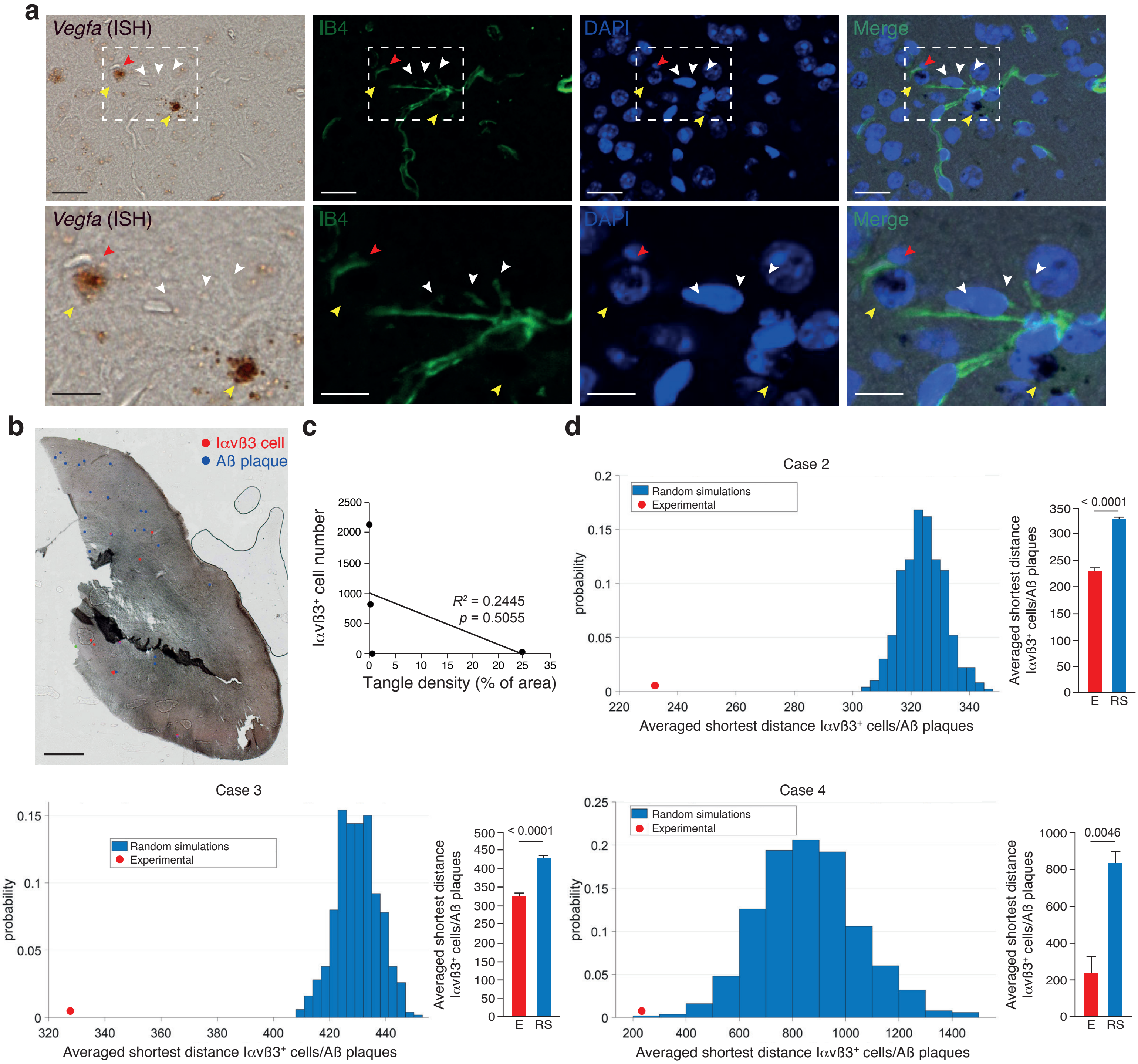

a
Vegfa (ISH)
IB4
DAPI
Merge
b
Iαvβ3 cell
Aβ plaque
c
Iαvβ3+ cell number
R^2 = 0.2445
p = 0.5055
Tangle density (% of area)
d
Case 2
Random simulations
Experimental
probability
Averaged shortest distance Iαvβ3+ cells/Aβ plaques
< 0.0001
E
RS
Case 3
Random simulations
Experimental
probability
Averaged shortest distance Iαvβ3+ cells/Aβ plaques
< 0.0001
E
RS
Case 4
Random simulations
Experimental
probability
Averaged shortest distance Iαvβ3+ cells/Aβ plaques
0.0046
E
RS

Supplementary Figure 1 | Angiogenesis is associated with Aß plaques in AD. a Cortical confocal projection from 8-month-old *APP-PSEN1/+* mice stained with *Vegfa* (ISH; brown), and endothelial (IB4; green) and nuclear (DAPI; blue) markers. Insets in the lower row show the dashed white rectangles depicted in the upper row. Yellow arrowheads point to cells with high *Vegfa* expression and white arrowheads indicate extruding filopodia from blood vessels. Scale bar = 20 µm in low and 10 µm in high magnification images. **b** A human hippocampal brain slice where the position of Integrin αvß3$^+$ –Iαvß3– cells (red dots) and Aß plaques (blue dots) are indicated. Scale bar = 1 mm. Green dots were used as landmarks. **c** Absence of correlation between Iαvß3$^+$ cells and the Thio-S positive tangle load. Spearman *r* correlation. **d** Upper and Lower rows: Left graph, representation of the probability of the averaged shortest distance between Iαvß3$^+$ cells and Aß plaques in 500 random simulations (RS, blue bars) where Aß plaques position was fixed and the Iαvß3$^+$ cells location was randomized. Red dots represent the experimental measurement. Right graph, quantification of the shortest distance between Iαvß3$^+$ cells and Aß plaques in an experimental (E) and in the first 10 random simulations (RS). Geodesic distances between Iαvß3$^+$ cells and nearest Aß plaques are presented as mean $\pm$ S.E.M. *n* are individual distances. Case 2: *n* = 825 (E) and 8250 (RS) Iαvß3$^+$ cells and 178 Aß plaques; Case 3: *n* = 2132 (E) and 21320 (RS) Iαvß3$^+$ cells and 356 Aß plaques; Case 4: *n* = 10 (E) and 100 (RS) Iαvß3$^+$ cells and 23 Aß plaques. Student's *t*-test.

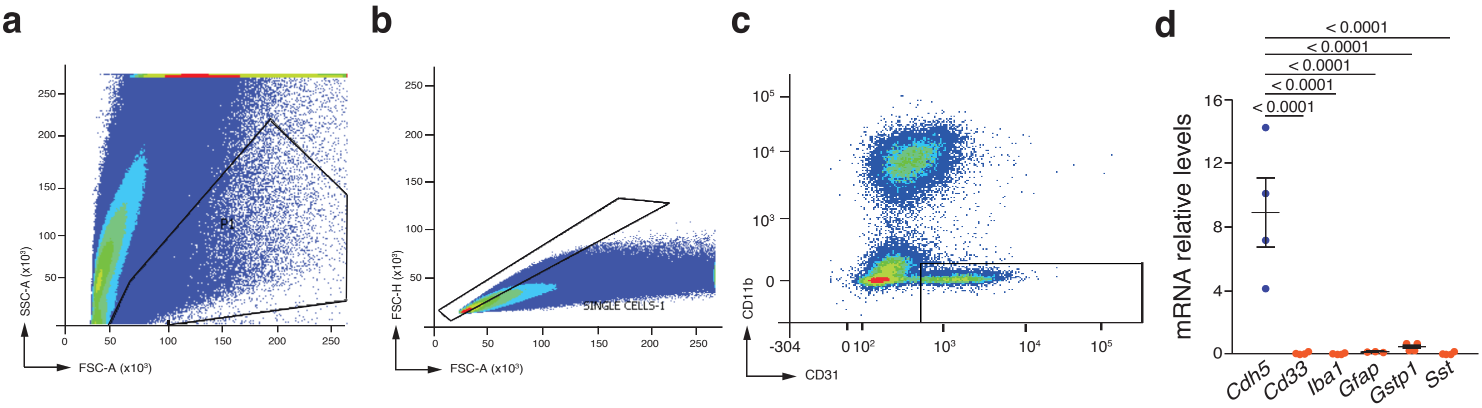

a
SSC-A (x10³)
FSC-A (x10³)
P1
b
FSC-H (x10³)
FSC-A (x10³)
SINGLE CELLS-1
c
CD11b
CD31
d
mRNA relative levels
< 0.0001
< 0.0001
< 0.0001
< 0.0001
< 0.0001
Cdh5
Cd33
Iba1
Gfap
Gstp1
Sst

Supplementary Figure 2 | Gating strategy to isolate endothelial cells from adult mice. a–c Fluorescence-activated cell sorting was performed according to guidelines[69] in contour density plots for isolation of endothelial cells in Fig. 3b, c. **a** Debris and dead cells were discarded by forward (FSC) and side (SSC) scatters dispersion of events. **b** Singlets of events were selected according to FSC height (FSC-H) versus area (FSC-A). **c** Endothelial cells were isolated using fluorescence-activated cell sorting from 18-month-old *APP-PSEN1/+* and wild-type mice using CD31 as a positive and CD11b as a negative marker. Every dot represents an event and isolated cells are those contained in the black rectangle. **d** *Cdh5* (vascular cadherin 5), *Cd33* and *Iba1* (microglia), *Gfap* (astrocytes), *Gstp1* (oligodendrocytes), and *Stt* (neuronal) mRNA levels were determined in isolated endothelial cells using qRT-PCR. *Hmbs* was used as housekeeping control (Mean ± SEM; n = 4 mice; ANOVA, post hoc Tukey's test).

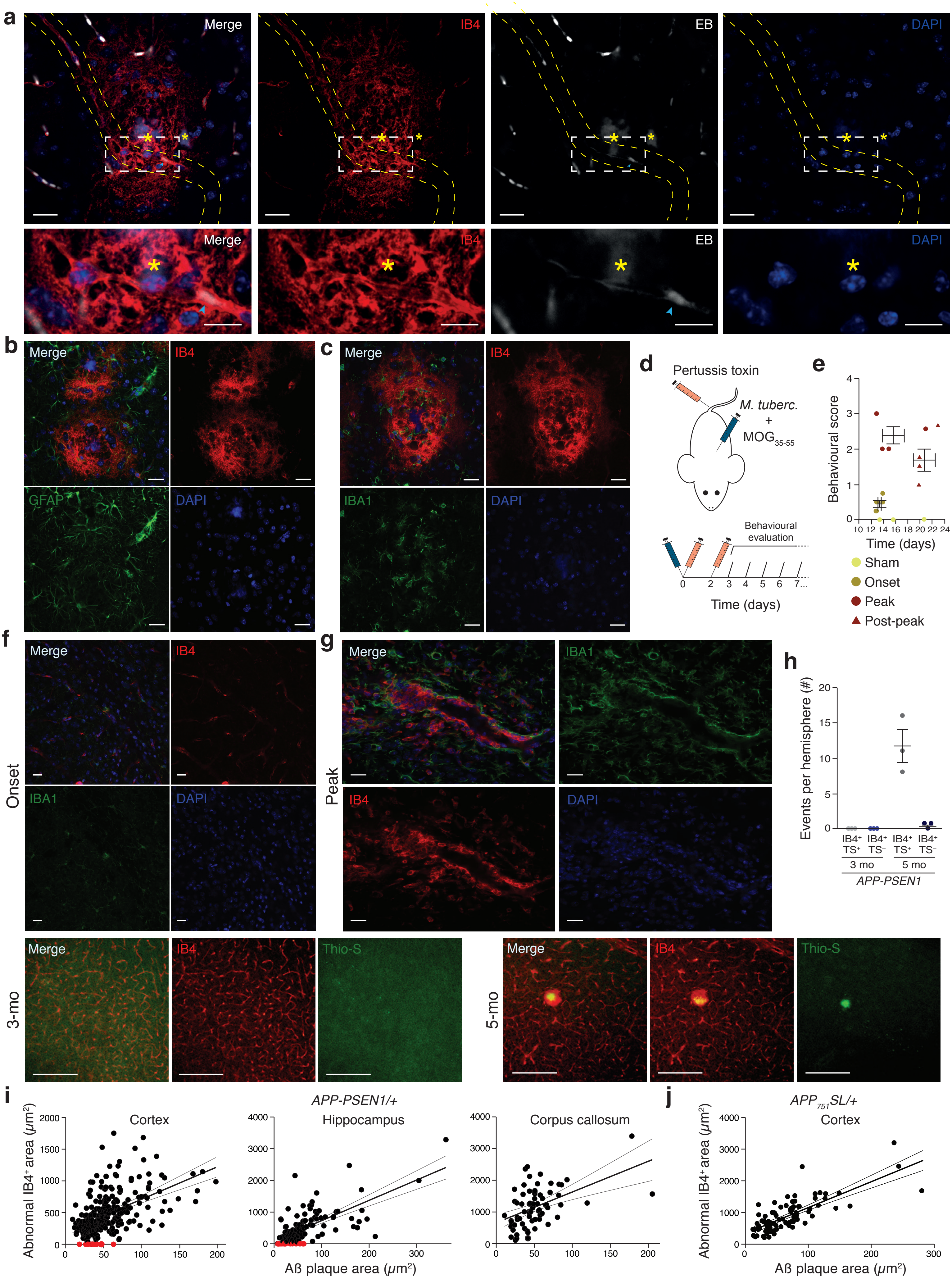

a
Merge
IB4
EB
DAPI
b
GFAP
c
IBA1
d
Pertussis toxin
M. tuberc.
+
MOG35-55
Behavioural evaluation
Time (days)
e
Behavioural score
Sham
Onset
Peak
Post-peak
f
Onset
g
Peak
h
Events per hemisphere (#)
IB4+ TS+
IB4+ TS−
3 mo
5 mo
APP-PSEN1
Thio-S
3-mo
5-mo
i
APP-PSEN1/+
Cortex
Hippocampus
Corpus callosum
Abnormal IB4+ area (μm2)
Aβ plaque area (μm2)
j
APP751SL/+

Supplementary Figure 3 | IB4⁺ vascular abnormalities. a Cortical confocal projection from 8-month-old *APP-PSEN1/+* mice injected with Evans Blue (EB, white) and stained with EC (IB4; red) and nuclear (DAPI; blue) markers. Yellow asterisks indicate Aß plaques. Lower row images show the rectangles depicted in the upper row images. Dashed yellow lines indicate a blood vessel and blue arrowheads point to a perfused (EB^+) vessel. Scale bars = 20 µm in low and 10 µm in high magnification images. **b**–**c** Absence of colocalization between vascular abnormalities and astrocytes ($GFAP^+$, **b**) or microglia ($IBA1^+$, **c**). Scale bars (**b**, **c**) = 20 µm. **d** Schematic representation of the experimental autoimmune encephalomyelitis (EAE) mouse model. $MOG_{35–55}$: Myelin Oligodendrocyte Glycoprotein, amino acids 30 to 55. *M. tuberc.*: *Mycobacterium tuberculosis*. **e** Behavioral score and the temporal data classified samples as onset, peak, and post-peak. Mean ± SEM. **f**–**g** Cortical confocal images at disease onset (**f**) and peak (**g**) stained with endothelial (IB4; red), microglial (IBA1, green), and nuclear (DAPI; blue) markers. Scale bar = 20 µm. **h** Upper row: Quantification of the number of events. TS: Thio-S; mo: -month-old. Mean ± SEM. *n* = 3 mice. Lower row: Images of cortical coronal brain slice from 3- (left panels row) and 5- (right panels) mo *$APP_{751}SL$/+* mice stained with endothelial (IB4; red), Aß (Thioflavin-S; Thio-S; green), and nuclear (DAPI; blue) markers. Scale bar = 200 µm. Right graph, quantification of the number of vascular abnormalities ($IB4^+$) and Aß plaques (Thioflavin-S –TS–$^+$). **i**, **j** Fit of Aß (Thio-S) plaque *versus* abnormal $IB4^+$ areas from 8- and 12-mo (**i**, *APP-PSEN1/+*) and 8-mo (**j**, *$APP_{751}SL$/+*) coronal brain sections stained as in Fig. 3e–i. Thick lines represent the fit and the thin lines the 95% confidence interval. Red points indicate plaques without associated abnormal $IB4^+$ areas. *APP-PSEN1/+*: Cortex: *n* = 263 plaques from 5 mice. r^2 =

0.2487; $y = 5.193x + 197.3$. Hippocampus: $n = 125$ plaques from 5 mice. $r^2 = 0.4672$; $y = 6.411x + 89.35$. Corpus callosum: $n = 69$ plaques from 5 mice. $r^2 = 0.2415$; $y = 9.7x + 660.7$. *$APP_{751}SL/+$*: Cortex: $n = 90$ plaques from 6 different mice. $r^2 = 0.5943$; $y = 4.107x + 360.1$. Spearman r with $p < 0.0001$ in all correlations.

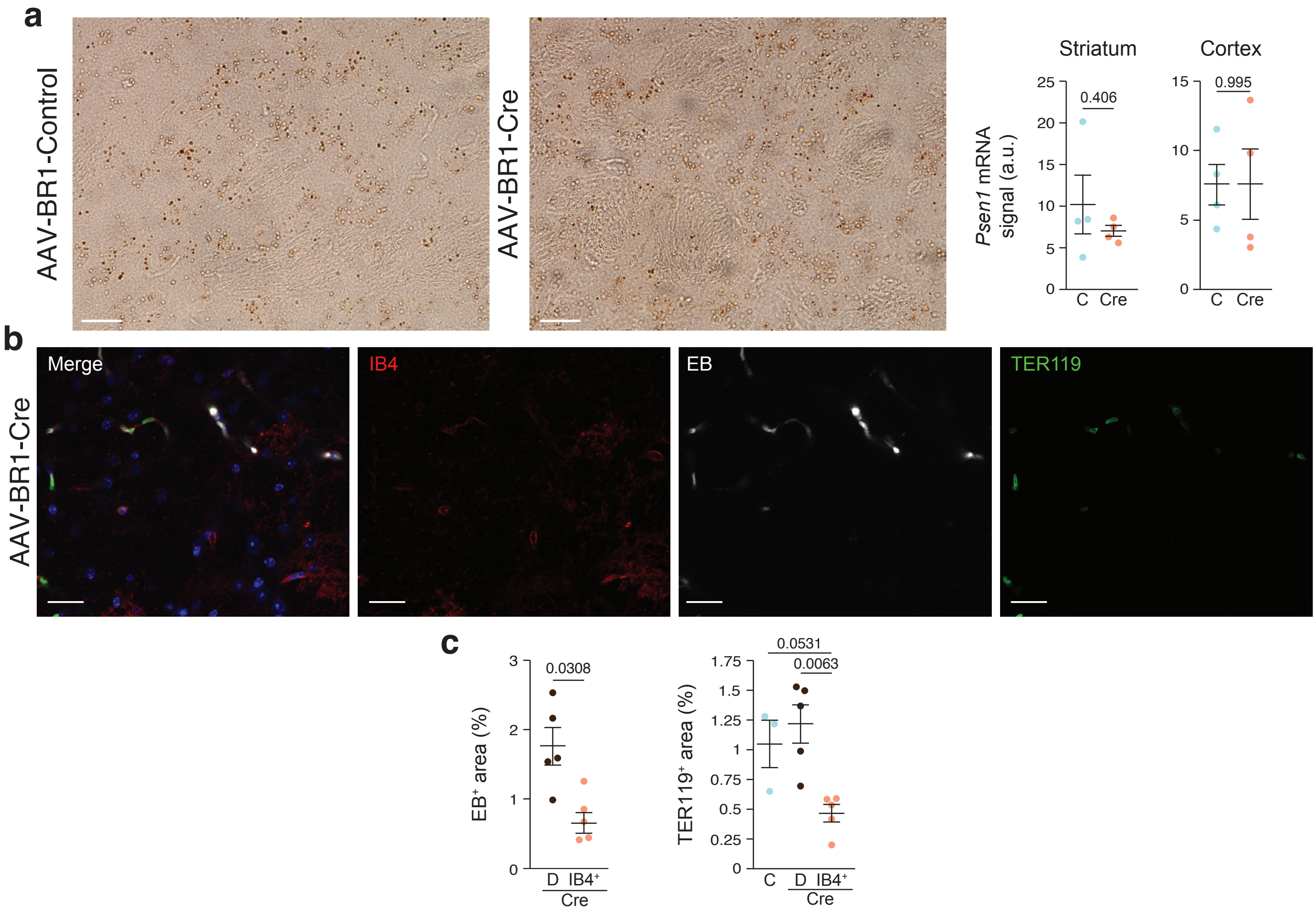
a
AAV-BR1-Control
AAV-BR1-Cre
Striatum
Cortex
Psen1 mRNA signal (a.u.)
0.406
0.995
C
Cre
b
AAV-BR1-Cre
Merge
IB4
EB
TER119
c
EB+ area (%)
0.0308
D
IB4+
Cre
TER119+ area (%)
0.0531
0.0063
C
D
IB4+
Cre

Supplementary Figure 4 | Reduced perfusion in abnormal IB4$^+$ areas produced by adult inhibition of endothelial γ-secretase activity. a Striatal confocal XY images from *Psen1*$^{loxP/loxP}$; *Psen2*$^{-/-}$ mice were injected with cerebral endothelium-specific adeno-associated control (AAV-BR1-Control; C) or Cre recombinase-expressing (AAV-BR1-Cre; Cre) viruses and stained with endothelial (IB4; red) and *Psen1* (*in situ hybridization*, ISH; brown) markers. Scale bar = 20 µm. Quantification of endothelial *Psen1*$^+$ signal in the striatum (left graph) and the cortex (right graph) of C (light blue dots) and Cre (orange dots) mice (a.u.: arbitrarty units). Mean ± SEM. *n* = 4 mice; Student's *t*-test. **b** Hippocampal slices from C and Cre mice perfused with Evans blue (EB; white) and stained with endothelial (IB4; red), red cells (TER119; green), and nuclear (DAPI; blue) markers. Scale bar = 40 µm. **c** Quantification of: left graph, percentage of area occupied by EB$^+$ area in distal vessels (D, brown dots) and in VaS (orange dots) from Cre mice. Mean ± SEM. *n* = 5; Student's *t*-test. Right graph: Percentage of area occupied by TER119$^+$ area in hippocampus form C and Cre (in distal –D– vessels and in VaS) mice. Mean ± SEM. *n* = 3 (C) and 5 (Cre) mice; ANOVA, post hoc Tukey's test.

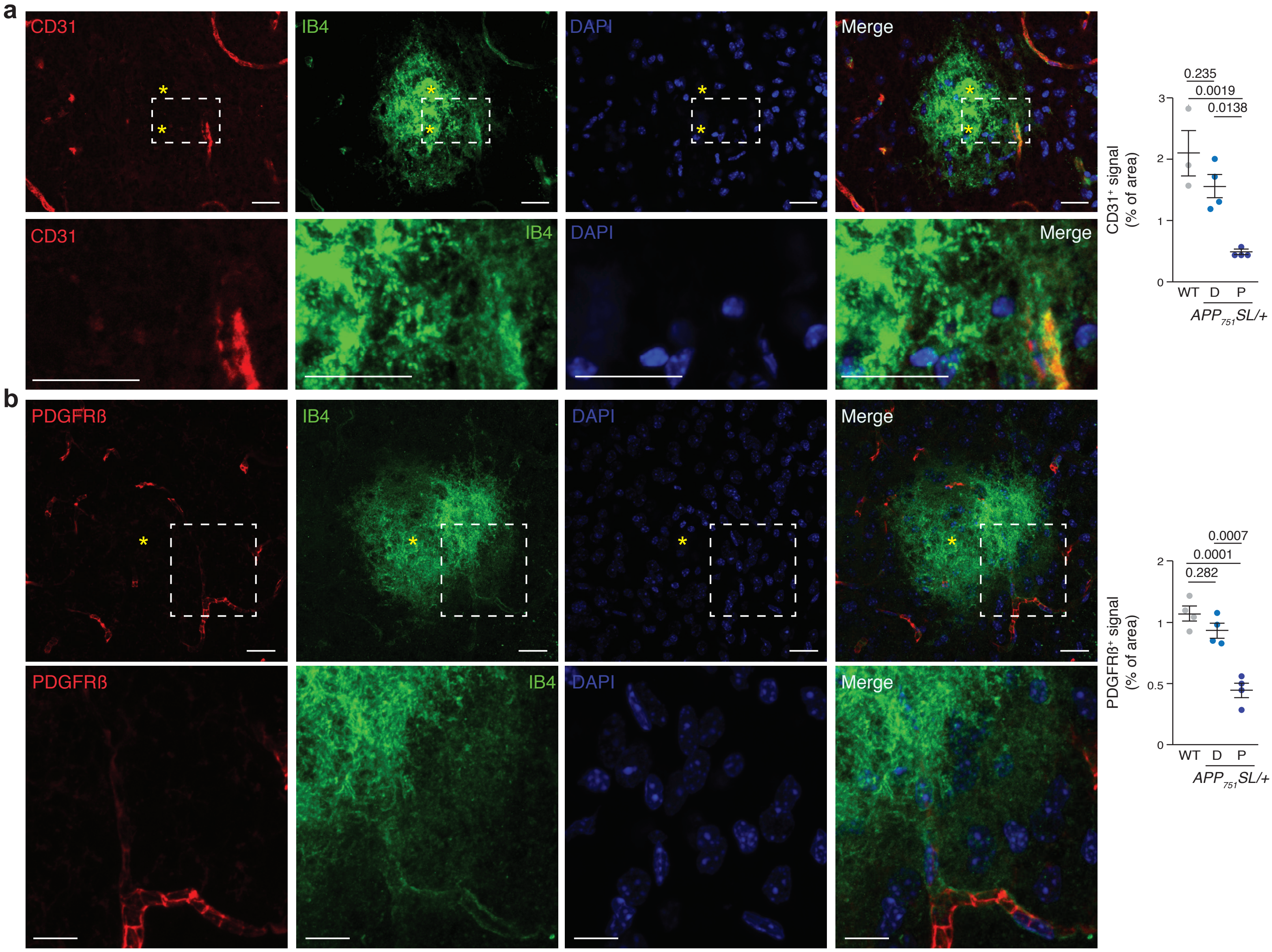

a
CD31
IB4
DAPI
Merge
0.235
0.0019
0.0138
CD31⁺ signal (% of area)
WT
D
P
APP751SL/+
b
PDGFRß
IB4
DAPI
Merge
0.0007
0.0001
0.282
PDGFRß⁺ signal (% of area)
WT
D
P
APP751SL/+

Supplementary Figure 5 | Vessel loss proximal to Aß plaques. a, **b** Cortical confocal projection of coronal brain slices from 8-month-old *APP-PSEN1/+* mice stained with (**a**) endothelial (CD31; red and IB4; green) and nuclear (DAPI; blue) markers or with (**b**) pericyte (PDGFRß; red), endothelial (IB4; green), and nuclear (DAPI; blue) markers. Aß plaques are indicated with yellow asterisks. Lower images are high magnification of dashed white rectangles. Scale bar = 20 µm. Right graphs are the quantification of the percentage of $CD31^{+}$ (**a**) or $PDGFRß^{+}$ (**b**) signal distal (D; light blue dots) and proximal (P; blue dots) to Aß plaques. Mean ± SEM. *n* = 4 ($APP_{751}SL/+$) mice; ANOVA, post hoc Tukey's test.

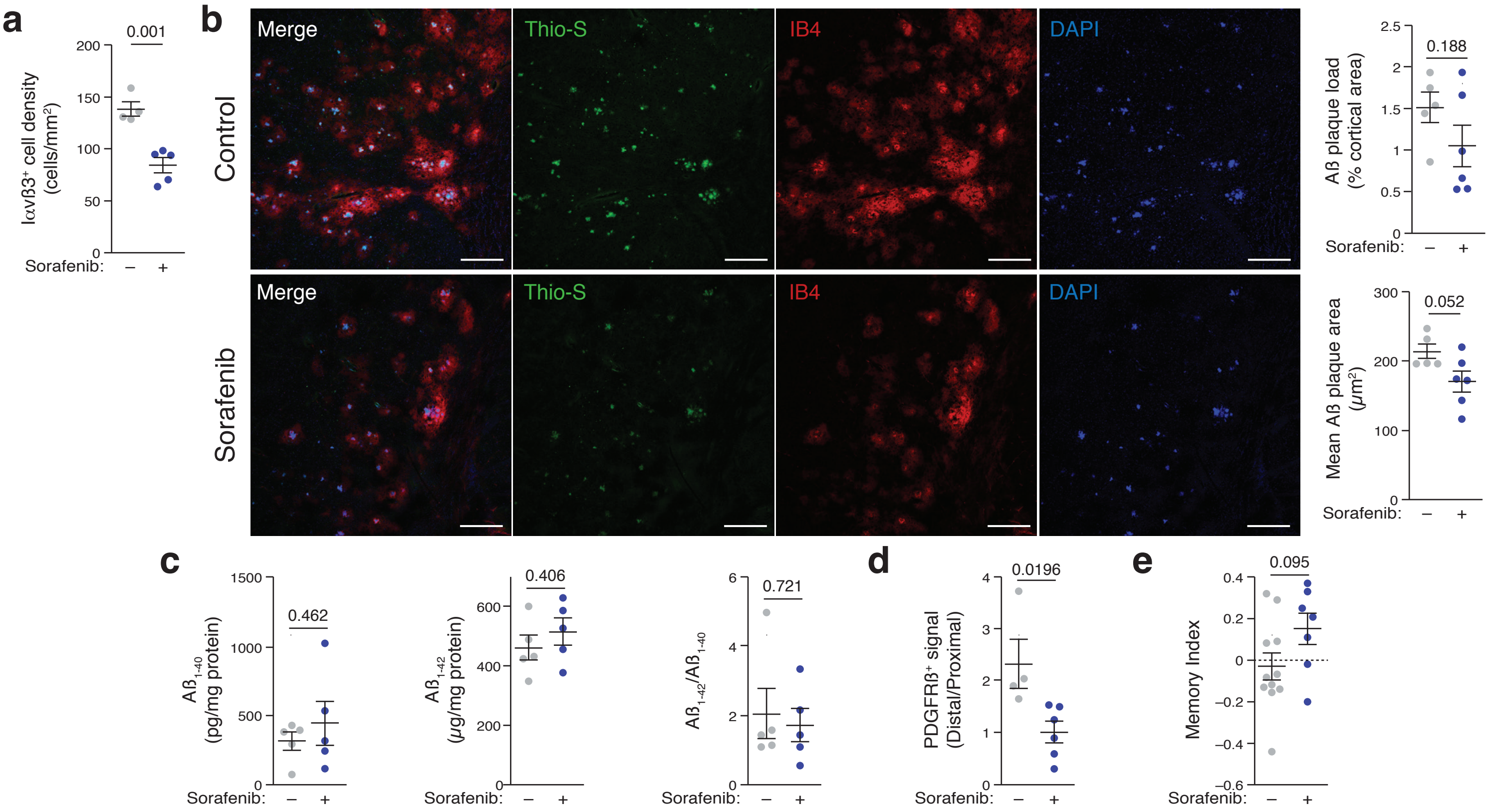
a
0.001
Iαvβ3+ cell density (cells/mm2)
Sorafenib: – +
b
Control
Sorafenib
Merge
Thio-S
IB4
DAPI
0.188
Aβ plaque load (% cortical area)
0.052
Mean Aβ plaque area (μm2)
Sorafenib: – +
c
0.462
Aβ1-40 (pg/mg protein)
0.406
Aβ1-42 (μg/mg protein)
0.721
Aβ1-42/Aβ1-40
d
0.0196
PDGFRβ+ signal (Distal/Proximal)
e
0.095
Memory Index
Sorafenib: – +

Supplementary Figure 6 | Inhibition of angiogenesis in an AD mouse model. **a**–**f** 7-month-old *$APP_{751}SL$/+* mice were treated with vehicle (–) or Sorafenib (+) for one month (30 mg/kg/2 day for 30 days). **a** Quantification of the Iαvß3^{+} cell density in Control (–; gray bar) and Sorafenib (+; blue bar) -treated mice. Mean ± S.E.M. *n* = 4 (–) or 5 (+) mice; Student's *t*-test. **b** Cortical slices stained with Aß (Thioflavin-S, Thio-S; green), endothelial/VaS (IB4; red), and nuclear (DAPI; blue) markers. Scale bar = 200 µm. Right graphs: Quantification of the Aß plaque load (upper graph) and mean Aß plaque area (lower graph). Mean ± S.E.M. *n* = 5 (–) or 6 (+) mice; Student's *t*-test. **c** $Aß_{1-40}$ (left graph), $Aß_{1-42}$ (centre graph), and $Aß_{1-42}/Aß_{1-40}$ ratio (right graph) determined by ELISA. Mean ± S.E.M. *n* = 5 (–) or 6 (+) mice; Student's *t*-test. **d** Quantification of the ration between PDGFRß$^{+}$ area distal and proximal to Aß plaques. Mean ± SEM. *n* = 4 (–) or *n* = 5 (+) mice; Student's *t*-test. **e** Short-term memory index in the novel object recognition test. Mean ± SEM. *n* = 11 (–) or *n* = 7 (+) mice; Student's *t*-test.

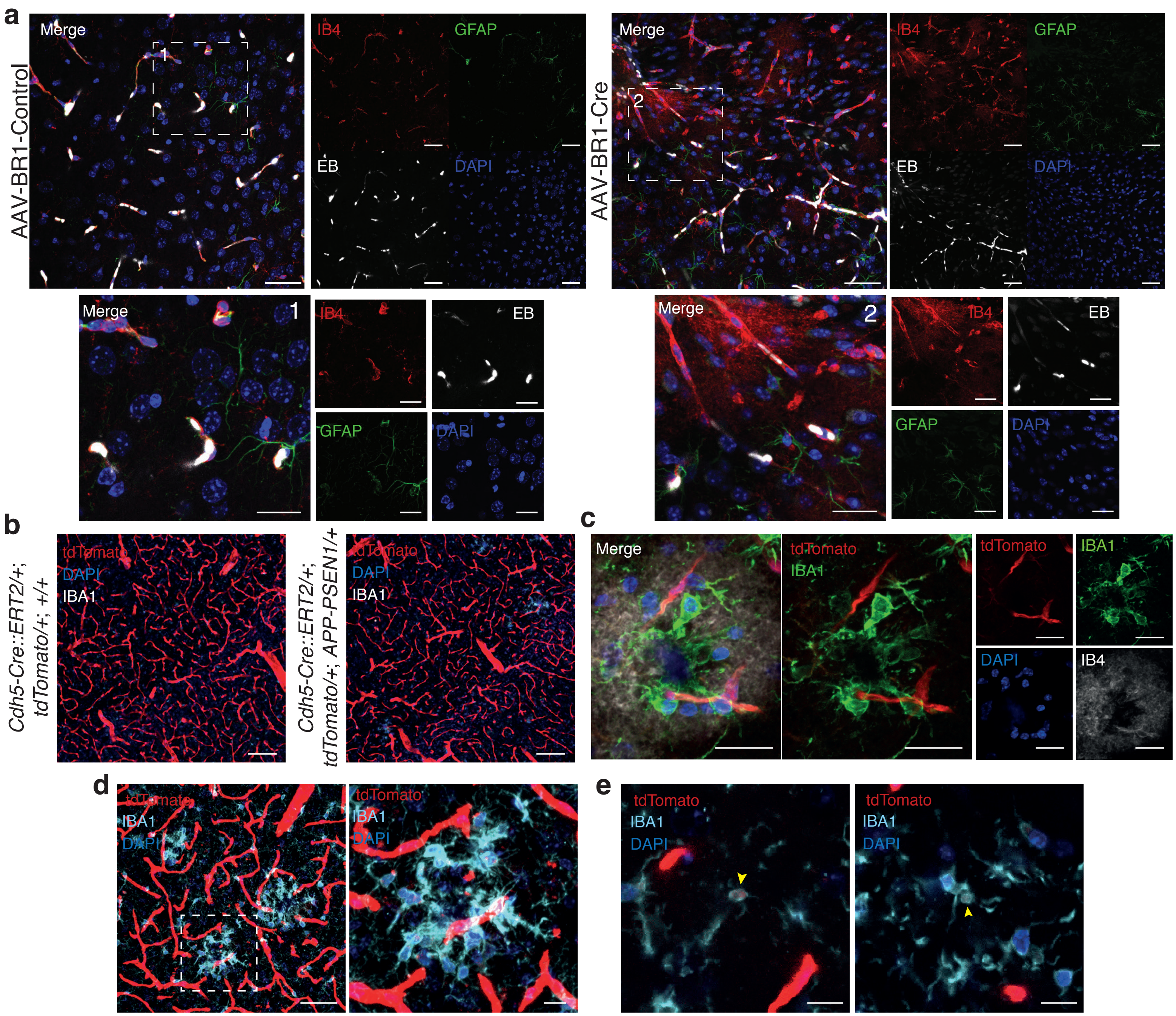

a
AAV-BR1-Control
Merge
1
IB4
GFAP
EB
DAPI
AAV-BR1-Cre
Merge
2
IB4
GFAP
EB
DAPI
b
Cdh5-Cre::ERT2/+; tdTomato/+; +/+
tdTomato
DAPI
IBA1
Cdh5-Cre::ERT2/+; tdTomato/+; APP-PSEN1/+
c
Merge
tdTomato
IBA1
DAPI
IB4
d
e

Supplementary Figure 7 | Astrocytes and microglial cells in VaS. a Astrocytes in a mouse model of adult inhibition of endothelial γ-secretase activity. Striatal confocal projections of coronal brain slices *Psen1*$^{loxP/loxP}$; *Psen2*$^{–/–}$ mice injected with AAV-BR1-Control (left) or AAV-BR1-Cre (right), and, two months later, perfused with Evans blue (EB; white) and stained with endothelial (IB4; red), astrocytic (GFAP; green), and nuclear (DAPI; blue) markers. Lower rows are high magnification of the dashed white rectangles depicted in the upper row. Scale bar = respectively 40 µm and 20 µm in low and high magnification images. **b**–**e** Microglial-blood vessel interaction proximal to Aß plaques. **b** Confocal images of coronal brain slices from 8-month-old *Cdh5-Cre::ERT2/*+; *R26-LSL-tdTomato/*+; +/+ (left panel) and *Cdh5-Cre::ERT2/*+; *R26-LSL-tdTomato/*+; *APP-PSEN1/*+ (**b**, right panel–**e**) tamoxifen-treated mice and stained with a tdTomato antibody (**c**) or direct tdTomato fluorescence (**b**, **d**, **e**; red) and with microglial (IBA1; cyan in **b**, **d**, **e**; green in **c**), endothelial (IB4; white in **c**), and nuclear (DAPI; blue) markers. Right images show the dashed white rectangles depicted in the left image in (**d**). **e** Microglial pouches containing tdTomato^{+} material are indicated with yellow arrowheads Scale bar = 100 µm in **b**; 20 µm in **c** and **e**; 50 µm and 10 µm in respectively low and high magnification images in **d**.
