# Supplementary material for: Non-productive angiogenesis disassembles Aß plaque-associated blood vessels
Source: Nat Commun. 2021 May 25;12:3098. doi: 10.1038/s41467-021-23337-z (PMC8149638; doi:10.1038/s41467-021-23337-z)
Supplement: Supplementary file 3 — Description of Additional Supplementary Files [file 41467_2021_23337_MOESM3_ESM.pdf]

Description of Additional Supplementary Files

File Name: Supplementary Data 1
Description: A description of the main characteristics of the samples used per experiment.

File Name: Supplementary Data 2
Description: Differentially expressed genes between *APP-PSEN1/+* and WT endothelial cells.

File Name: Supplementary Data 3
Description: Gene set enrichment analysis (GSEA) between *APP-PSEN1/+* and WT endothelial cells.

File Name: Supplementary Movie 1
Description: iDisco analysis of an *APP-PSEN1/+* brain stained with the vascular/VaS marker IB4.

File Name: Supplementary Movie 2
Description: Z-stack of the confocal projection presented in Figure 6a.

File Name: Supplementary Movie 3
Description: 3D reconstruction of the confocal projection presented in Figure 6a.

File Name: Supplementary Movie 4
Description: 3D reconstruction of the confocal projection presented in Figure 6c.

File Name: Supplementary Movie 5
Description: 3D reconstruction of a confocal projection similar to the one presented in Figure 6c.

File Name: Supplementary Movie 6
Description: 3D reconstruction of the confocal projection presented in SFigure 7d.

File Name: Supplementary Movie 7
Description: 3D reconstruction of a confocal projection similar to the one presented in Figure 6c.

File Name: Supplementary Movie 8
Description: 3D reconstruction of the confocal projection presented in Figure 6d.

File Name: Supplementary Movie 9
Description: 3D reconstruction of a confocal projection similar to the one presented in Figure 6d.

File Name: Supplementary Movie 10
Description: Z-stack of a confocal projection similar to the one presented in Figure 6d.
