# Supplementary material for: Non-productive angiogenesis disassembles Aß plaque-associated blood vessels
Source: Nat Commun. 2021 May 25;12:3098. doi: 10.1038/s41467-021-23337-z (PMC8149638; doi:10.1038/s41467-021-23337-z)
Supplement: Supplementary file 17 — Reporting Summary [file 41467_2021_23337_MOESM17_ESM.pdf]

nature research

Corresponding author(s): Pascual, Alberto

Last updated by author(s): Mar 28, 2021

Reporting Summary

Nature Research wishes to improve the reproducibility of the work that we publish. This form provides structure for consistency and transparency in reporting. For further information on Nature Research policies, see Authors & Referees and the Editorial Policy Checklist.

Statistics

For all statistical analyses, confirm that the following items are present in the figure legend, table legend, main text, or Methods section.

n/a	Confirmed	
☐	☒	The exact sample size (*n*) for each experimental group/condition, given as a discrete number and unit of measurement
☐	☒	A statement on whether measurements were taken from distinct samples or whether the same sample was measured repeatedly
☐	☒	The statistical test(s) used AND whether they are one- or two-sided<br>*Only common tests should be described solely by name; describe more complex techniques in the Methods section.*
☐	☒	A description of all covariates tested
☐	☒	A description of any assumptions or corrections, such as tests of normality and adjustment for multiple comparisons
☐	☒	A full description of the statistical parameters including central tendency (e.g. means) or other basic estimates (e.g. regression coefficient) AND variation (e.g. standard deviation) or associated estimates of uncertainty (e.g. confidence intervals)
☐	☒	For null hypothesis testing, the test statistic (e.g. *F*, *t*, *r*) with confidence intervals, effect sizes, degrees of freedom and *P* value noted<br>*Give P values as exact values whenever suitable.*
☒	☐	For Bayesian analysis, information on the choice of priors and Markov chain Monte Carlo settings
☒	☐	For hierarchical and complex designs, identification of the appropriate level for tests and full reporting of outcomes
☒	☐	Estimates of effect sizes (e.g. Cohen's *d*, Pearson's *r*), indicating how they were calculated

Our web collection on statistics for biologists contains articles on many of the points above.

Software and code

Policy information about availability of computer code

Data collection	Not data collection software was used
Data analysis	Fiji (v. 2.0.0)<br>FACSDiva (v. 8.0 Becton Dickinson)<br>R statistical processing environment (v.3.6.3 RStudio)<br>LIMMA (v.3.1 Bioconductor)<br>GraphPad Prism version 6.0 (GraphPad)<br>Expression Console software (v.4.0 Affymetrix)<br>Imaris software (Bitplane)<br>ImspectorPro Software (LaVision BioTec)

For manuscripts utilizing custom algorithms or software that are central to the research but not yet described in published literature, software must be made available to editors/reviewers. We strongly encourage code deposition in a community repository (e.g. GitHub). See the Nature Research guidelines for submitting code & software for further information.

Data

Policy information about availability of data

All manuscripts must include a data availability statement. This statement should provide the following information, where applicable:

- Accession codes, unique identifiers, or web links for publicly available datasets
- A list of figures that have associated raw data
- A description of any restrictions on data availability

The raw data of endothelial cells microarray are available at the Gene Expression Omnibus under accession code GSE121729 (https://www.ncbi.nlm.nih.gov/geo/query/acc.cgi?acc=GSE121729). Source data are provided with this paper as a Source Data File.

Field-specific reporting

Please select the one below that is the best fit for your research. If you are not sure, read the appropriate sections before making your selection.

☒ Life sciences ☐ Behavioural & social sciences ☐ Ecological, evolutionary & environmental sciences

For a reference copy of the document with all sections, see nature.com/documents/nr-reporting-summary-flat.pdf

Life sciences study design

All studies must disclose on these points even when the disclosure is negative.

Sample size	No sample calculations were performed. The number used was selected based in previous publications in the field.
Data exclusions	No data were excluded
Replication	All the experiments were replicated in biological samples and no repetition was excluded from the study. All replications attempts were successful. A minimal of three independents experiments were performed.
Randomization	Allocation was random
Blinding	The investigators were blinded to group allocation during data collection and/or analysis.

Reporting for specific materials, systems and methods

We require information from authors about some types of materials, experimental systems and methods used in many studies. Here, indicate whether each material, system or method listed is relevant to your study. If you are not sure if a list item applies to your research, read the appropriate section before selecting a response.

Materials & experimental systems

n/a	Involved in the study	
☐	☒	Antibodies
☒	☐	Eukaryotic cell lines
☒	☐	Palaeontology
☐	☒	Animals and other organisms
☐	☒	Human research participants
☒	☐	Clinical data

Methods

n/a	Involved in the study	
☒	☐	ChIP-seq
☐	☒	Flow cytometry
☒	☐	MRI-based neuroimaging

Antibodies

Antibodies used

Alexa-488 and Alexa-568-conjugated streptavidin, Jackson, 1:500
Alexa-488, Alexa-568, Alexa-647-conjugated streptavidin (Jackson, 1:500)
Alexa-488, Alexa-568, and Alexa-647, Invitrogen, 1:400 or Molecular Probes, 1:500
anti-AQP4 (Sigma, 1:5,000, A5971)
anti-CD11b-APC (eBioscience, 1:200, 17-0112)
anti-CD140b (PDGFR-ß) (APB5), PE, eBioscience™ (Invitrogen, 1:200, 12-1402-81)
anti-CD16/CD32 (BD Bioscience, 1:200, clone 2.462, 553142)
anti-CD31 (PECAM-1) e Bioscience, (clone 390, Invitrogen, 1:500, 11-0311-82)
anti-CD68 (Bio-Rad, 1:100, MCA1957GA)
anti-GFAP (Sigma, 1:1,000, G3893)
anti-I[illegible]vß3 (Abcam, 1:50, ab7166)
anti-IBA1 (Wako, 1:400, 01-1974)
anti-Laminin (Sigma, 1:250, L9393)
anti-mCherry (EnCor Biotechnology, 1:1000, CPCA-mCherry)
anti-TER119 e Bioscience (Invitrogen, 1:400, 14-5921-85)
Isolectin B4, biotin conjugated (Sigma, 1:50, L2140)

Validation

Reference 35 from the main text for: Isolectin B4, anti-Laminin, anti-IBA1, anti- PDGFR-ß.
Reference 23 from the main text for: anti-GFAP, anti-CD16/CD32, anti-CD140b and anti-CD11b.
anti-CD31 cited in PMID: 32094452
anti-Ter119 cited in PMID: 28276839
Reference 32 from the main text for anti-I[illegible]vß3
anti-AQP4 cited in PMID: 24943270

anti-CD68 cited in PMID: 3312248

Animals and other organisms

Policy information about studies involving animals; ARRIVE guidelines recommended for reporting animal research

Laboratory animals

Mouse (Described in STABLE 1):
Figure 1 Figure 2. Human samples used in this study Figure 3 Figure 4 Figure 5 Figure 6 SFigure 3 SFigure 4 SFigure 5 SFigure 6
Panel 1B Panel 3A Age Sex Genotype Treatment Panel A Age Sex Genotype Panel D-G Panel A Panel A Panel A, B, D
Age Sex Genotype Braak & Braak SEX Age at exitus PMI Age Sex Genotype 3mo ♀ PSEN1Flox/Flox; PSEN2–/– AAV-BR1-CRE Age Sex Genotype 7mo ♀ Cdh5-CRE::ERT2/+; APP-PSEN1/+; R26LSL-tdTomato/+ Age Sex Genotype EAE stage Age Sex Genotype Treatment Age Sex Genotype Age Sex Genotype
14mo ♂ APP-PSEN1/+ 4 ♂ 79 4 h 18mo ♀ APP-PSEN1/+ 3mo ♀ PSEN1Flox/Flox; PSEN2–/– AAV-BR1-Control 8mo ♂ C57J 7mo ♀ Cdh5-CRE::ERT2/+; APP-PSEN1/+; R26LSL-tdTomato/+ 7 weeks ♀ C57J Onset 3mo ♀ PSEN1Flox/Flox; PSEN2–/– AAV-BR1-CRE 8mo ♂ APP751SL/+ 8mo ♀ APP751SL/+
14mo ♂ APP-PSEN1/+ 4 ♂ 85 8 h 18mo ♂ APP-PSEN1/+ 3mo ♀ PSEN1Flox/Flox; PSEN2–/– AAV-BR1-Control 8mo ♂ C57J 7mo ♀ Cdh5-CRE::ERT2/+; +/+; R26LSL-tdTomato/+ 7 weeks ♀ C57J Onset 3mo ♀ PSEN1Flox/Flox; PSEN2–/– AAV-BR1-Control 8mo ♀ APP751SL/+ 8mo ♀ APP751SL/+
14mo ♀ APP-PSEN1/+ 5 ♀ 82 na 18mo ♂ APP-PSEN1/+ 3mo ♂ PSEN1Flox/Flox; PSEN2–/– AAV-BR1-CRE 8mo ♂ C57J 7mo ♀ Cdh5-CRE::ERT2/+; APP-PSEN1/+; R26LSL-tdTomato/+ 7 weeks ♀ C57J Onset 3mo ♀ PSEN1Flox/Flox; PSEN2–/– AAV-BR1-Control 8mo ♀ APP751SL/+ 8mo ♀ APP751SL/+
6 ♀ 97 na 18mo ♀ C57J 3mo ♂ PSEN1Flox/Flox; PSEN2–/– AAV-BR1-Control 8mo ♂ C57J 7mo ♀ Cdh5-CRE::ERT2/+; +/+; R26LSL-tdTomato/+ 7 weeks ♀ C57J Onset 3mo ♂ PSEN1Flox/Flox; PSEN2–/– AAV-BR1-CRE 8mo ♂ APP751SL/+ 8mo ♀ APP751SL/+
Panel 1E 6 ♂ 92 6 h 18mo ♂ C57J 3mo ♂ PSEN1Flox/Flox; PSEN2–/– AAV-BR1-CRE 8mo ♀ APP751SL/+ 7mo ♀ Cdh5-CRE::ERT2/+; +/+; R26LSL-tdTomato/+ 7 weeks ♀ C57J Onset 3mo ♂ PSEN1Flox/Flox; PSEN2–/– AAV-BR1-Control 8mo ♂ C57J 8mo ♀ APP751SL/+
Age Sex Genotype 1 ♀ 48 8 h 18mo ♂ C57J 3mo ♂ PSEN1Flox/Flox; PSEN2–/– AAV-BR1-Control 8mo ♀ APP751SL/+ 7mo ♂ Cdh5-CRE::ERT2/+; APP-PSEN1/+; R26LSL-tdTomato/+ 7 weeks ♀ C57J Peak 3mo ♂ PSEN1Flox/Flox; PSEN2–/– AAV-BR1-CRE 8mo ♂ C57J 8mo ♀ APP751SL/+
8mo ♂ C57J 0 ♂ 59 6 h 3mo ♂ PSEN1Flox/Flox; PSEN2–/– AAV-BR1-CRE 8mo ♀ APP751SL/+ 7 weeks ♀ C57J Peak 3mo ♂ PSEN1Flox/Flox; PSEN2–/– AAV-BR1-Control 8mo ♂ C57J 8mo ♂ APP751SL/+
8mo ♂ C57J 0 ♂ 68 7 h Panel 3D 3mo ♂ PSEN1Flox/Flox; PSEN2–/– AAV-BR1-CRE 8mo ♂ APP751SL/+ 7 weeks ♀ C57J Peak 3mo ♂ PSEN1Flox/Flox; PSEN2–/– AAV-BR1-CRE 8mo ♂ APP751SL/+
8mo ♀ C57J 0 ♀ 56 2 h Age Sex Genotype 3mo ♂ PSEN1Flox/Flox; PSEN2–/– AAV-BR1-Control 8mo ♂ APP751SL/+ 7 weeks ♀ C57J Peak Panel B 8mo ♂ APP751SL/+
8mo ♀ C57J 1 ♀ 67 na 8mo ♀ APP751SL/+ 3mo ♀ PSEN1Flox/Flox; PSEN2–/– AAV-BR1-CRE 7 weeks ♀ C57J Post-peak Panel B Age Sex Genotype 8mo ♂ APP751SL/+
8mo ♀ C57J 8mo ♀ APP751SL/+ 3mo ♀ PSEN1Flox/Flox; PSEN2–/– AAV-BR1-Control Panel B 7 weeks ♀ C57J Post-peak Age Sex Genotype Treatment 8mo ♀ APP751SL/+ 8mo ♀ APP751SL/+
8mo ♂ APP-PSEN1/+ PMI: Postmortem intervale 8mo ♂ APP751SL/+ 3mo ♂ PSEN1Flox/Flox; PSEN2–/– AAV-BR1-CRE Age Sex Genotype 7 weeks ♀ C57J Post-peak 3mo ♂ PSEN1Flox/Flox; PSEN2–/– AAV-BR1-CRE 8mo ♀ APP751SL/+
8mo ♂ APP-PSEN1/+ na: not available 8mo ♀ C57J 3mo ♂ PSEN1Flox/Flox; PSEN2–/– AAV-BR1-Control 8mo ♂ C57J 7 weeks ♀ C57J Post-peak 3mo ♂ PSEN1Flox/Flox; PSEN2–/– AAV-BR1-Control 8mo ♀ APP751SL/+ Panel C
8mo ♂ APP-PSEN1/+ 8mo ♀ C57J 3mo ♂ PSEN1Flox/Flox; PSEN2–/– AAV-BR1-CRE 8mo ♀ C57J 7 weeks ♀ C57J Sham 3mo ♀ PSEN1Flox/Flox; PSEN2–/– AAV-BR1-CRE 8mo ♂ APP751SL/+ Age Sex Genotype
8mo ♀ APP-PSEN1/+ 8mo ♂ C57J 3mo ♂ PSEN1Flox/Flox; PSEN2–/– AAV-BR1-CRE 8mo ♂ C57J 7 weeks ♀ C57J Sham 3mo ♀ PSEN1Flox/Flox; PSEN2–/– AAV-BR1-Control 8mo ♂ APP751SL/+ 8mo ♂ APP751SL/+
8mo ♀ APP-PSEN1/+ 8mo ♀ C57J 5mo ♀ PSEN1Flox/Flox; PSEN2–/– AAV-BR1-CRE 8mo ♂ C57J 7 weeks ♀ C57J Sham 3mo ♂ PSEN1Flox/Flox; PSEN2–/– AAV-BR1-CRE 8mo ♂ C57J 8mo ♂ APP751SL/+
5mo ♀ PSEN1Flox/Flox; PSEN2–/– AAV-BR1-Control 8mo ♂ APP751SL/+ 3mo ♂ PSEN1Flox/Flox; PSEN2–/– AAV-BR1-Control 8mo ♀ C57J 8mo ♂ APP751SL/+
Panel 3G 5mo ♂ PSEN1Flox/Flox; PSEN2–/– AAV-BR1-CRE 8mo ♀ APP751SL/+ Panel H 3mo ♂ PSEN1Flox/Flox; PSEN2–/– AAV-BR1-CRE 8mo ♂ C57J 8mo ♂ APP751SL/+
Panel 1G Age Sex Genotype 5mo ♂ PSEN1Flox/Flox; PSEN2–/– AAV-BR1-Control 8mo ♀ APP751SL/+ Age Sex Genotype 3mo ♂ PSEN1Flox/Flox; PSEN2–/– AAV-BR1-CRE 8mo ♂ C57J 8mo ♀ APP751SL/+
Age Sex Genotype 8mo ♀ C57J 8mo ♂ APP751SL/+ 5mo ♀ APP751SL/+ 5mo ♀ PSEN1Flox/Flox; PSEN2–/– AAV-BR1-CRE 8mo ♂ APP751SL/+
8mo ♂ C57J 8mo ♀ C57J 5mo ♀ APP751SL/+ 5mo ♀ PSEN1Flox/Flox; PSEN2–/– AAV-BR1-Control 8mo ♀ APP751SL/+
8mo ♂ C57J 8mo ♂ C57J Panel C 5mo ♀ APP751SL/+ 5mo ♂ PSEN1Flox/Flox; PSEN2–/– AAV-BR1-CRE 8mo ♀ APP751SL/+
8mo ♀ C57J 8mo ♂ APP-PSEN1/+ Age Sex Genotype 3mo ♀ APP751SL/+ 5mo ♂ PSEN1Flox/Flox; PSEN2–/– AAV-BR1-Control 8mo ♂ APP751SL/+
8mo ♀ C57J 8mo ♂ APP-PSEN1/+ 8mo ♂ C57J 3mo ♂ APP751SL/+ 8mo ♀ APP751SL/+
8mo ♂ APP-PSEN1/+ 8mo ♂ APP-PSEN1/+ 8mo ♂ C57J 3mo ♀ APP751SL/+
8mo ♂ APP-PSEN1/+ 12mo ♂ APP-PSEN1/+ 8mo ♂ C57J Panel E
8mo ♂ APP-PSEN1/+ 12mo ♂ APP-PSEN1/+ 8mo ♂ C57J Panel I Age Sex Genotype
8mo ♀ APP-PSEN1/+ 12mo ♂ APP-PSEN1/+ 8mo ♂ APP751SL/+ Age Sex Genotype 8mo ♂ APP751SL/+

8mo ♀ APP-PSEN1/+ 8mo ♂ APP751SL/+ 8mo ♀ APP751SL/+ 8mo ♂ APP-PSEN1/+ 8mo ♂ APP751SL/+
8mo ♀ APP751SL/+ 8mo ♀ APP751SL/+ 8mo ♂ APP-PSEN1/+ 8mo ♂ APP751SL/+
mo: -month-old 8mo ♀ APP751SL/+ 8mo ♀ APP751SL/+ 8mo ♂ APP-PSEN1/+ 8mo ♂ APP751SL/+
8mo ♀ APP751SL/+ 12mo ♂ APP-PSEN1/+ 8mo ♀ APP751SL/+
8mo ♂ APP751SL/+ 12mo ♂ APP-PSEN1/+ 8mo ♂ APP751SL/+
8mo ♂ APP751SL/+ 12mo ♂ APP-PSEN1/+ 8mo ♀ APP751SL/+
8mo ♀ APP751SL/+
Panel 3K Panel J 8mo ♂ APP751SL/+
Age Sex Genotype Age Sex Genotype 8mo ♀ APP751SL/+
5mo ♀ +/+; CBF1-Venus 8mo ♂ APP751SL/+ 8mo ♂ APP751SL/+
5mo ♀ +/+; CBF1-Venus 8mo ♀ APP751SL/+ 8mo ♀ APP751SL/+
5mo ♀ APP751SL/+; CBF1-Venus 8mo ♀ APP751SL/+ 8mo ♀ APP751SL/+
5mo ♀ APP751SL/+; CBF1-Venus 8mo ♀ APP751SL/+ 8mo ♀ APP751SL/+
4mo ♀ APP751SL/+; CBF1-Venus 8mo ♂ APP751SL/+ 8mo ♀ APP751SL/+
4mo ♂ +/+; CBF1-Venus 8mo ♂ APP751SL/+ 8mo ♀ APP751SL/+
4mo ♀ APP751SL/+; CBF1-Venus 8mo ♀ APP751SL/+
4mo ♂ APP751SL/+; CBF1-Venus 8mo ♂ APP751SL/+
4mo ♂ APP751SL/+; CBF1-Venus 8mo ♀ APP751SL/+
8mo ♀ APP751SL/+
8mo ♂ APP751SL/+
8mo ♀ APP751SL/+
8mo ♀ APP751SL/+
8mo ♂ APP751SL/+
8mo ♂ APP751SL/+
8mo ♂ APP751SL/+

Wild animals

No wild animals were used in the study

Field-collected samples

No field collected samples were used in the study

Ethics oversight

Housing and treatments were performed according to the animal care guidelines of European Community Council (86/60/EEC). Principles of laboratory animal care (NIH publication No. 86-23, revised 1985) were followed, as well as specific Spanish national laws where applicable. The competent Spanish authority approved all the procedures ("Consejería de agricultura, pesca y desarrollo rural. Dirección general de la producción agrícola y ganadera").

Note that full information on the approval of the study protocol must also be provided in the manuscript.

Human research participants

Policy information about studies involving human research participants

Population characteristics

Describe the covariate-relevant population characteristics of the human research participants (e.g. age, gender, genotypic information, past and current diagnosis and treatment categories). If you filled out the behavioural & social sciences study design questions and have nothing to add here, write "See above."

Recruitment

Describe how participants were recruited. Outline any potential self-selection bias or other biases that may be present and how these are likely to impact results.

Ethics oversight

Identify the organization(s) that approved the study protocol.

Note that full information on the approval of the study protocol must also be provided in the manuscript.

Flow Cytometry

Plots

Confirm that:

☒ The axis labels state the marker and fluorochrome used (e.g. CD4-FITC).

☒ The axis scales are clearly visible. Include numbers along axes only for bottom left plot of group (a 'group' is an analysis of identical markers).

☒ All plots are contour plots with outliers or pseudocolor plots.

☒ A numerical value for number of cells or percentage (with statistics) is provided.

Methodology

Sample preparation	Endothelial cells isolation method was modified from a previously described of microglial isolation(10). Briefly, mice were anesthetised and transcardially perfused with HBSS (–CaCl2/–MgCl2) (Gibco) and cortices were dissected and then dissociated using a Tissue Chopper (Vibratome, 800 series). Chemical digestion was then performed with a mix of papain (Worthington) (8 U/mL) and DNAse I (SIGMA; 80 Kunitz units/mL) followed by a Percoll gradient (GE healthcare) at 90 % in PBS (v/v). Cells were stained with primary conjugated monoclonal antibodies CD11b-APC (eBioscience) and CD31-PE (BD Bioscience) diluted 1:200 at 4°C for 30 min. Staining with isotype control-PE and isotype control-APC (eBioscience) at 1:200 dilution was used as negative control. Both control and experimental samples were simultaneously incubated with anti-CD16/CD32 (e-Bioscience) blocker antibody at 1:200. Cells were washed and sorted using a FACS Aria Fusion (Becton Dickinson) flow cytometer and data acquired and analysed with FACSDiva software 8.0 (Becton Dickinson).
Instrument	FACS Aria Fusion (Becton Dickinson)
Software	FACSDiva software 8.0 (Becton Dickinson)
Cell population abundance	Purity was determined by qRT-PCR of sorted cells using several markers.
Gating strategy	Gating strategy and data analysis were made according to guidelines(1). Debris and dead cells were discarded by forward and side scatter pattern. FSC-A and FSCH events distribution was used to gate single cells (Supplementary Fig. S4). Endothelial cells were identified as positive events for CD31 and negative for CD11b relative to negative controls. Percentages are relative to total single cells.

☒ Tick this box to confirm that a figure exemplifying the gating strategy is provided in the Supplementary Information.
